# Supplementary figures and images for: Diverse mutant selection windows shape spatial heterogeneity in evolving populations
Source: PLoS Comput Biol. 2024 Feb 22;20(2):e1011878. doi: 10.1371/journal.pcbi.1011878 (PMC10914271; doi:10.1371/journal.pcbi.1011878)

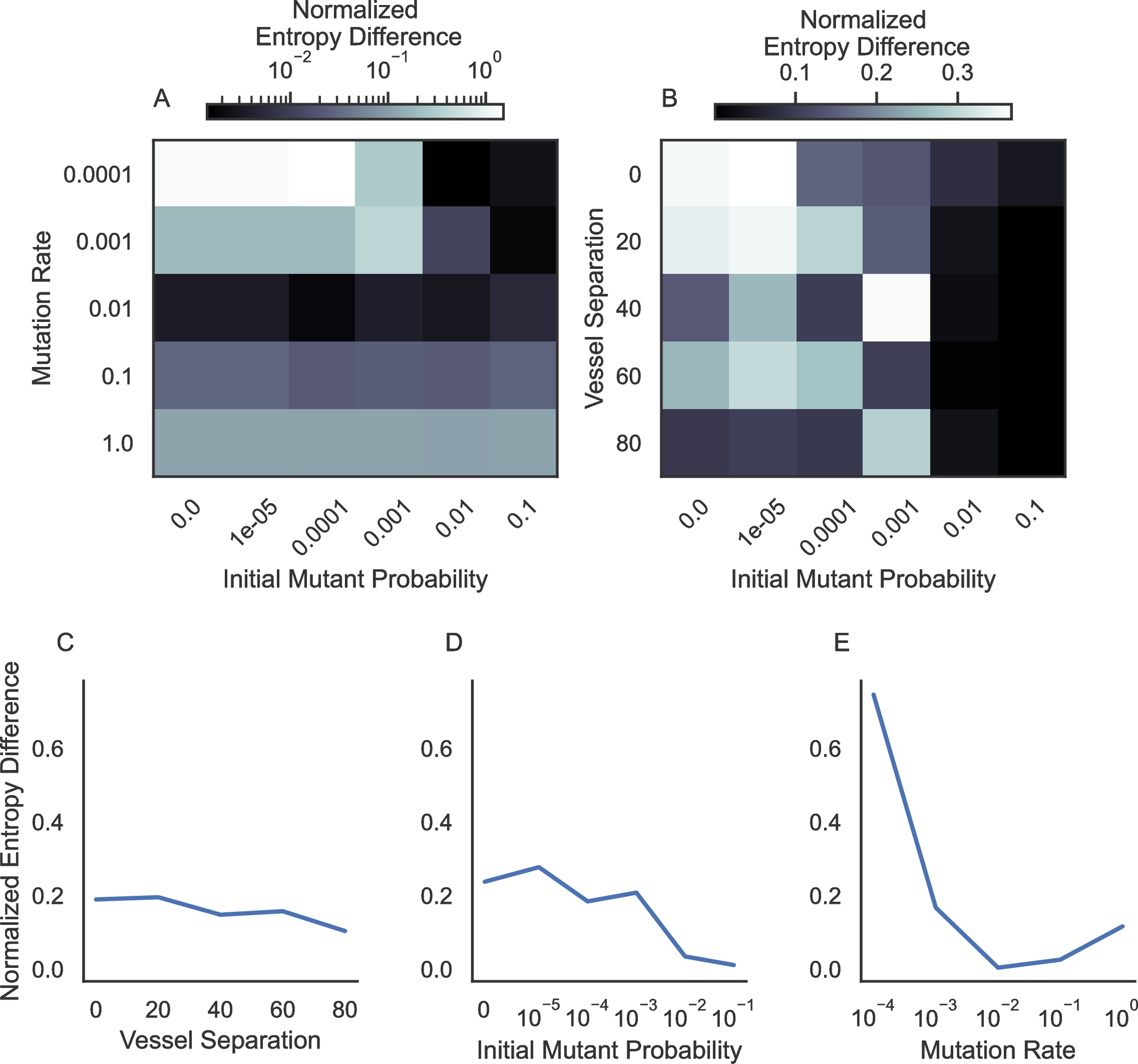

Supplement: S1 Fig — (A) Joint heatmap of mutation rate versus initial mutant probability. Normalized entropy difference is the squared difference between the Altieri entropy of the MSW map and the Altieri entropy of the final population distribution, normalized by the MSW entropy (Eq (6)). (B) Joint heatmap of vessel separation (lattice points between the two blood vessels) versus initial mutant probability. (C) Marginal distribution of normalized entropy difference versus blood vessel separation. (D) Marginal distribution of normalized entropy difference versus initial mutant probability. (E) Marginal distribution of normalized entropy difference versus mutation rate. (TIF) [file pcbi.1011878.s001.tif]

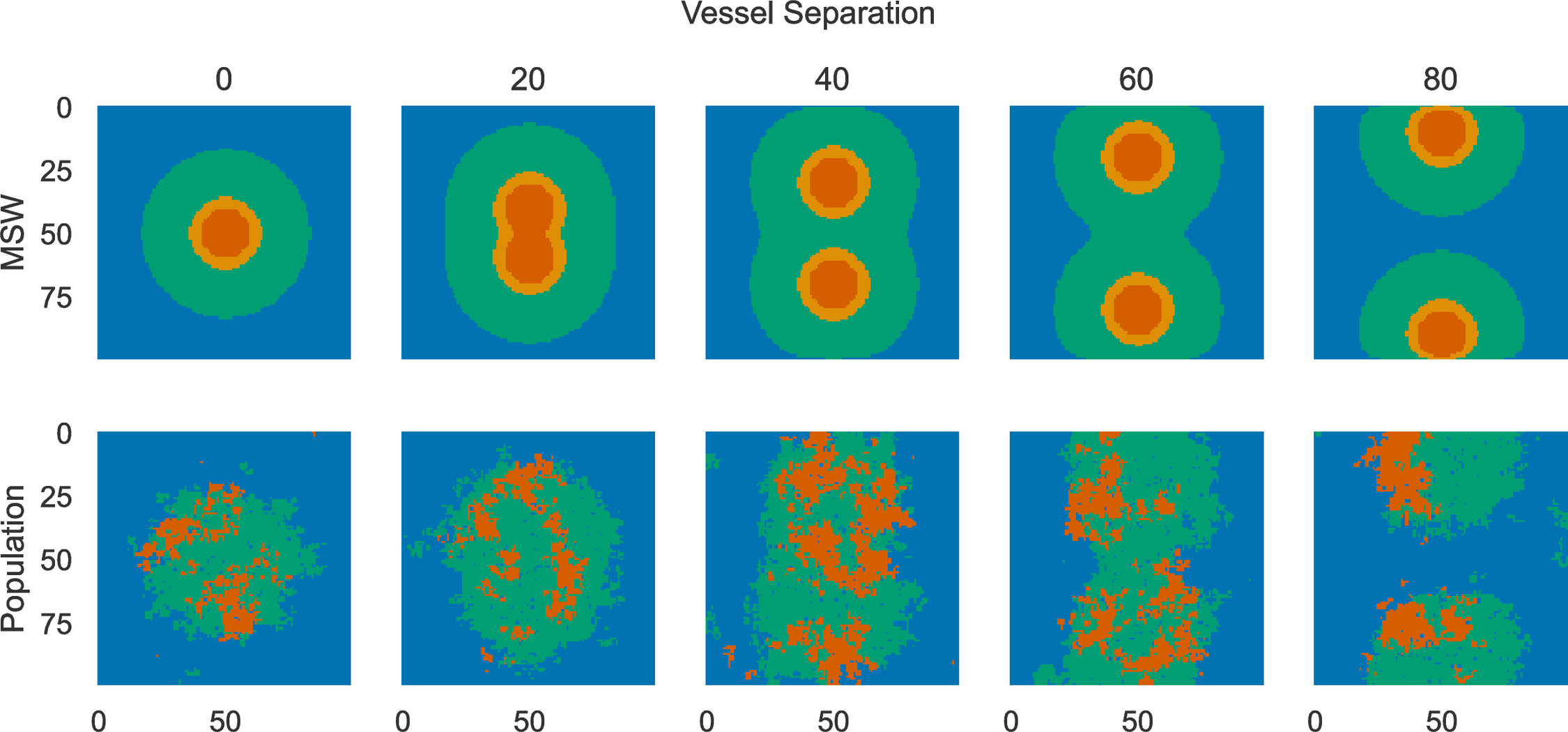

Supplement: S2 Fig — Each column corresponds to a different blood vessel separation distance labeled at the top of each column. Distance is in units of lattice points. Drug elimination rate γ = 0.01, mutation rate = 0.001, and initial mutant probability = 0.01. (TIF) [file pcbi.1011878.s002.tif]

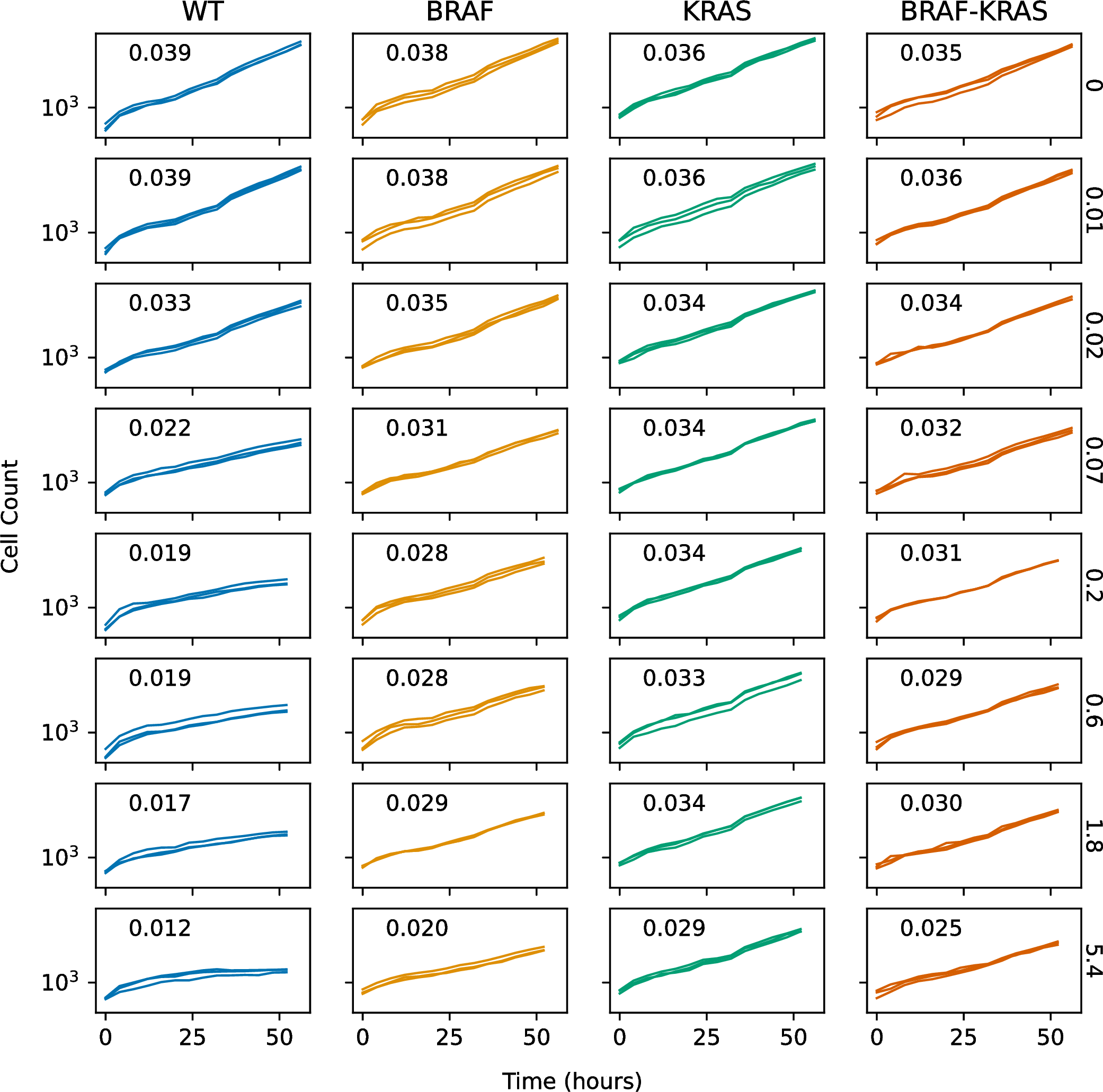

Supplement: S3 Fig — Each column corresponds to a cell type (WT, BRAF, KRAS, or BRAF-KRAS). Each row corresponds to a concentration of gefitinib (labeled on the right hand side in μM). Each condition has 3 replicates. Each condition is labeled with the estimated average growth rate (hr−1). (TIF) [file pcbi.1011878.s003.tif]

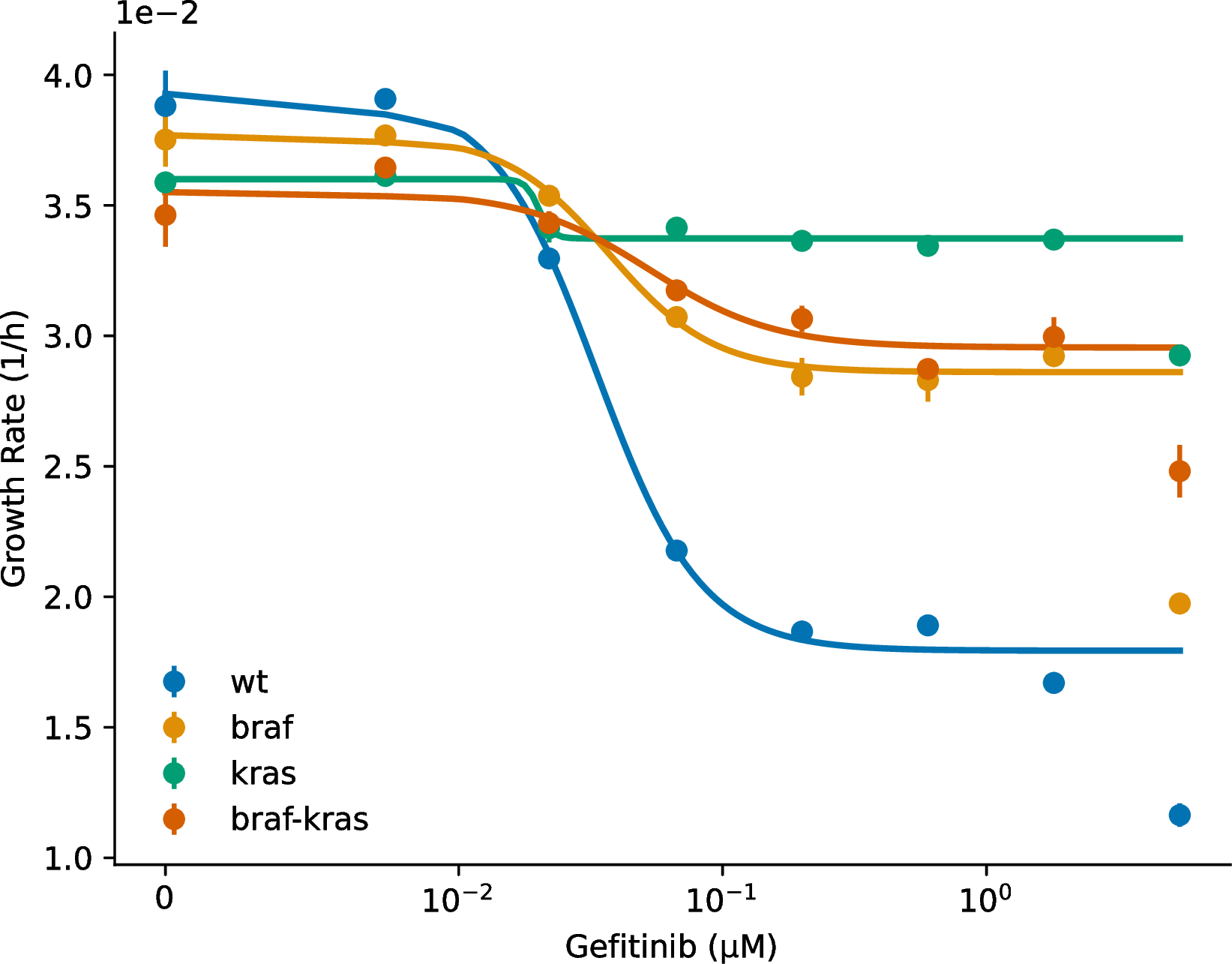

Supplement: S4 Fig — Growth rate versus drug concentration calculated from the data in S3 Fig. (TIF) [file pcbi.1011878.s004.tif]

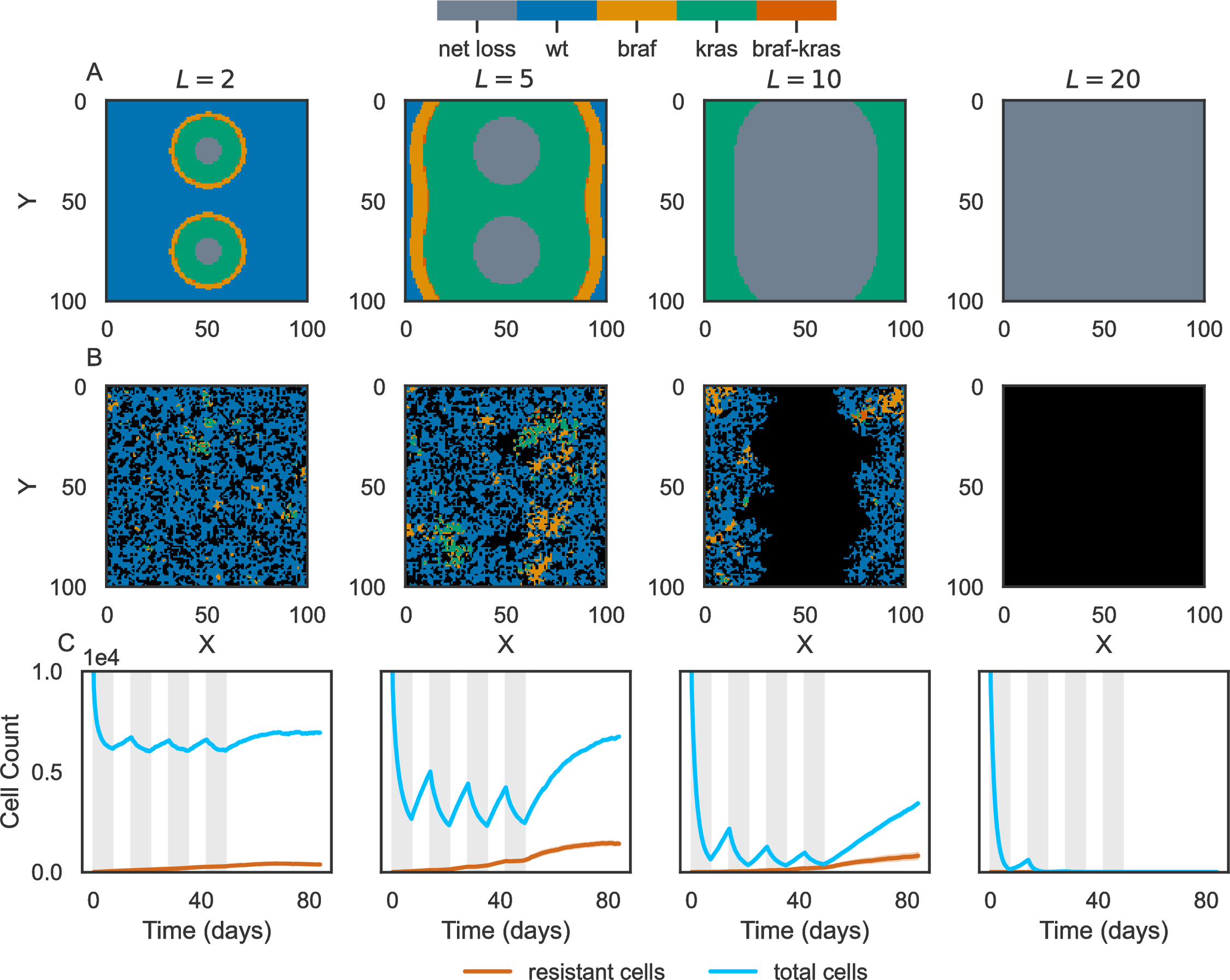

Supplement: S5 Fig — Summary of tumor therapy simulations with no pre-existing heterogeneity. Each column corresponds to a different drug diffusion characteristic length L. (A) Mutant selection window plots for different length scales resulting from drug diffusion from two blood vessels. The net loss regime represents a drug concentration that completely inhibits cell division. (B) Example simulations corresponding to the characteristic length in A. Black grid points indicate no cells at that position. (C) Average timecourse of total number of cells (blue) and the number of drug resistant cells (orange). Drug resistant refers to any cell that is not wild-type. Traces represent the average of N = 10 simulations and are shaded by the standard error. In many cases, the standard error is less than the width of the plot line. Gray vertical bars indicate the “drug on” time. (TIF) [file pcbi.1011878.s005.tif]
