# Supplementary material for: Diverse mutant selection windows shape spatial heterogeneity in evolving populations
Source: PLoS Comput Biol. 2024 Feb 22;20(2):e1011878. doi: 10.1371/journal.pcbi.1011878 (PMC10914271; doi:10.1371/journal.pcbi.1011878)
Supplement: Attachment — Submitted filename: Review___PLoS_Comp_Bio___Fitness_seascapes_promote_genetic_heterogeneity_through_spatiotemporally_distinct_mutant_selection_windows.pdf [file pcbi.1011878.s006.pdf]

Review 'Fitness seascapes promote genetic heterogeneity through spatiotemporally distinct mutant selection windows'

June 12, 2023

In this manuscript, the authors place the concept of mutation selection windows (MSWs) in the context of fitness landscapes and seascapes. They focus on the point that a mutant's relative fitness can be highly temporally or spatially dependent and demonstrate this using a simplified model that combines pharmacokinetics and randomly drawn dose-response curves. This is a simple point, but nonetheless one worth considering in depth, especially given that drug treatments have no choice but to occur in 3D space and in time. The manuscript is more or less well-written. However, it is quite spare (with only two figures, and relatively short text/analysis), and falls short of what I would consider sufficient depth or novelty as it is written now. However, I will highlight areas in which I believe the manuscript can be improved, should the authors decide to do so.

My primary concern is that the manuscript is simultaneously terse and unfocused, and I am unsure what the central argument or novel insight is. Moreover, clarity is an issue. Central terms should be much more carefully defined and should reflect common understanding – for instance, in my experience, 'seascapes' typically refer specifically to time varying fitness landscapes, whereas the manuscript describes temporally varying as well as spatially dependent but stationary conditions. This is not so problematic in and of itself since this is primarily a semantic issue, but it contributes to my sense that the manuscript lacks a clear sense of focus. While nothing is explicitly wrong, per se, I am left wondering what I am supposed to conclude or learn. As I noted, and as the authors themselves note in the conclusion, many others have demonstrated that space and time are relevant for drug resistance evolution. Therefore, this is not a sufficiently novel point on its own. If instead, for instance, the manuscript focused specifically on temporally varying *or* spatially varying scenarios alone, and did so in a way that delved into the problem of drug resistance evolution in detail in that specific context, many more interesting and novel insights might be gleaned. Moreover, if the authors presented this in a way that highlighted the generalizability of their results (for instance, by describing what time or length-scales are relevant for drug resistance mutants to emerge with high probability), that would be really great and would certainly be of broad interest.

When it comes to the specifics of the modeling and the assumptions that are made, I see many opportunities for improvement. For instance, dynamics, initial conditions and stochasticity are all important factors in the process of mutation and selection, and yet they are never discussed in detail. Typically, the detailed dynamics of individuals and their positions in the spatial context of a drug gradient will determine the emergence of resistance mutants. Instead, the models discussed are highly simplified and the relevant calculations are limited to ones that are relatively well-known. I am left yearning for more depth in this regard. What happens in more complicated geometries than the ones described? These are almost certainly the relevant scenario in real tissue. What happens when the system is inherently dynamical and spatial, like when there is flow in a blood vessel? This can be highly relevant, as several pathogens are bloodborne rather than tissue resident. It is well-appreciated that evolutionary outcomes can be highly contingent on the initial spatial distribution of WT and mutant individuals. How does this play into these types of models? The final and most crucial point is that despite there the presence of highly heterogeneous MSWs in space and time, depending on the relevant length and time scales, this heterogeneity might be 'averaged over' on the relevant length or time scale of growth. Moreover, demographic stochasticity (e.g. genetic drift) might play a much more relevant role than anything related to the deterministic effects of selection. In what parameter regimes should I expect these to be the relevant phenomenology? These are all important considerations for the broader applicability of this type of modeling, and all of these points (especially this last point about the relevant domain of applicability) should be engaged with in earnest in both the analysis and discussion.

The abstract notes that this work highlights the 'utility' of this framework for evolutionary medicine, but I am not sure I see how such an abstract and theoretical model would be utilized in a clinical context. One of the main points of reference in the current manuscript is to Das et al (2018). The Das et al paper primarily discusses the concept of MSWs in a well-mixed setting. Importantly, in Das et al (2018), they explicitly connect this idea to data. What about data in the context of spatially or temporally fluctuating drug concentrations? With the advent of spatially resolved measurements, it is certainly plausible that one might be able to measure some of the relevant spatial gradients that are described. Perhaps the exactly appropriate dataset does not exist yet, but it is sure to come soon. It would be important to address this and to look for relevant data that might constrain the described models or, at the very least, propose what the appropriate experiment might look like given current technologies.

Finally, what is the broader relevance? There are certainly many well-established scenarios or conceptual touchstones on which temporal and spatial heterogeneity are a relevant perturbation, but none in particular are highlighted. It would perhaps be most effective to relate these MSW models to other theoretical concepts. One particularly relevant concept is that of fitness valley crossing. It is natural to ask, for instance, what is the simplest scenario in which temporally or spatially heterogeneous drug concentrations will significantly change the probability of fitness valley crossing? And more specifically, by what degree

does heterogeneity amplify or restrain fitness valley crossing? This is only one possible example, but it is important that the authors explicitly place this work in a broader context. As it is, it is unclear where this work exactly fits.

I have critiqued but also provided avenues for improvement of this manuscript. I believe if the authors come back with significant effort, either utilizing some of my suggestions (which are far from the space of all possibilities) or otherwise, this could be a suitable manuscript for publication in PLoS Computational Biology, especially since this topic is interesting. However, the manuscript does not deliver on this potential and requires quite significant structural changes and additional content.
