# Supplementary material for: Diverse mutant selection windows shape spatial heterogeneity in evolving populations
Source: PLoS Comput Biol. 2024 Feb 22;20(2):e1011878. doi: 10.1371/journal.pcbi.1011878 (PMC10914271; doi:10.1371/journal.pcbi.1011878)
Supplement: Attachment — Submitted filename: kingPlosComp2023review.pdf [file pcbi.1011878.s007.pdf]

Summary of the research

In this study the authors put forth a theoretical model for analyzing the dynamics of drug resistance in a pharmacological context, by examining mutant selection widows (MSWs) – the window of drug concentration in which a resistant phenotype has a higher fitness – embedded in fitness seascapes – an extension of a fitness landscape where a genotype's fitness may depend on external factors such as drug concentration. They show how multiple MSWs can be constructed if many resistant types exist, and how active selection windows can change with space and time.

The work is presented clearly, both in the main text as well as the figures.

I believe this work to be useful and of interest to the scientific community, however I have a number of issues which I think should be addressed.

Issues requiring attention

Major issues

- In describing the time-variation of MSWs the authors model the variation of drug concentration using a 1-compartment pharmacokinetic model. A description of what parameter values were taken (and why) is missing. Are these based on known values of a particular drug, or typical ranges? A reference here to relevant experimental literature (i.e. with measured k_elim and k_abs) would solidify this result.
- When investigating the spatial dynamics of MSWs the authors derive a steady state solution for the drug diffusion, which they then use to identify different MSW's in space. To obtain this steady state their driving equation (eq. 1) has the property of a constant influx of drug over time. How realistic is this assumption, as opposed to for example a delta spike at t=0 or some step function? While the constant flux is clearly necessary to obtain a nonzero steady state, I wonder to what extent this solution is still relevant to the biological problem, considering that in reality to drug would only be introduced for a limited duration. Perhaps if shown that this steady state is reached quickly compared to the duration of the drug injection, there might be a timeframe where it is relevant; but if the quantities of interest are the MSWs, that timeframe should be long enough for selection to act within the different types. Is there some argument for why this constant flux is realistic? Or, is there some way to show (e.g. through numerical solutions out of equilibrium) that the steady state MSW's capture the important behavior?

Minor issues

- Figure 1: In B and C the authors show 3 regimes: "wild type selection", "mutant selection" and "net loss". While the wild type and mutant selection regimes are clearly described as the regime where that type presents the higher growth rate, it is not explained what the "net loss" regime is.
- While the authors have done a good job of explaining their own work and results, both in the main text and through clear figures, I found that some background information was lacking, in particular towards readers who are potentially not familiar with all the concepts brought up. I found I needed to read Ref. 7 (Das et al., eLife, 2020) to find definitions of some of the terms used here. While there is of course nothing wrong with referring readers to references for detailed explanations, I believe some minor additions here can greatly improve the readability of the manuscript for readers not already closely familiar with this field.

- Line 28: There is no explanation of what is meant by “adjacent genotypes”. From ref. 7, I gathered this refers to “genotypes that differ by one mutation”. Some definition or explanation here would be appreciated.
- Line 49: The authors describe pairing each genotype with a random dose-response curve. While this is accompanied by some references, I think the authors could explain a bit more what this conceptually means, i.e. how a resistant strain would enter the tradeoff of taking a lower drug-free growth rate for a higher IC50. Furthermore, the functional form of the dose-response curves is missing, which I strongly feel should be included here (or in the methods section).
- Line 50: “IC50”, this technical term could use a definition
- Line 112: the “1-compartment pharmacokinetic model” could use a reference to point unfamiliar readers toward its derivation.
