# Supplementary material for: Diverse mutant selection windows shape spatial heterogeneity in evolving populations
Source: PLoS Comput Biol. 2024 Feb 22;20(2):e1011878. doi: 10.1371/journal.pcbi.1011878 (PMC10914271; doi:10.1371/journal.pcbi.1011878)
Supplement: Attachment — Submitted filename: response_letter.pdf [file pcbi.1011878.s008.pdf]

Taussig Cancer Institute
Cleveland Clinic
**Jacob G. Scott, MD, DPhil (Oxon)**

Dear Dr. Wodarz and Dr. Komarova,

Thank you for your thoughtful review of our manuscript, "Fitness seascapes promote genetic heterogeneity through spatiotemporally distinct mutant selection windows". The comments from the reviewers have helped strengthen the novelty, rigor, and impact of our work. Based on the reviewers feedback, we narrowed our analysis to focus on space only and included stochastic spatial simulations to understand how mutant selection windows shape evolution. We also clarified the language and added a box to help with terminology.

We respond to each of the reviewers specific concerns below. The reviewer comments are **bolded**. Our responses are directly below each comment.

**Reviewer 1**:

1. **No particular infectious disease has been considered as an application.**

   We thank the reviewer for their helpful and insightful feedback. In this work, we sought to maintain a general outlook that can be applied to a variety of disease states in evolutionary medicine, including infectious disease and cancer. As such, we avoided parameterizing our model with a specific disease in mind. Instead, we included a sensitivity analysis which explores the parameter regimes where MSWs may drive the evolution of drug resistance.

2. **The implications of the findings on drug administration treatment strategies have not been discussed.**

   Optimizing dosing strategies is a natural extension of this work. Indeed, our related work touches on this topic (King et al, 2022). While a formal exploration of dosing optimization is outside the scope of this manuscript, we have added potential future directions to the discussion.

3. **Also, while the effects of the spatial distribution of the drug concentration are important, and this is indeed an important novelty aspect of the present work, the results shown in Figure 2 are expected. Thus, I would suggest to incorporate an additional example, with a more complex blood vessel topography, that preferably comes from a certain infection; this would strengthen the computational aspect of the work as well as the usefulness of the model in treatment strategies.**

   This is an important point, and one that has been explored by our group in related work (Scott 2016). In revising this manuscript, we sought to explore stochastic spatial simulations in our relatively simple blood vessel arrangement as an initial experiment. We studied one aspect of blood vessel geometry by varying the distance between blood vessels and analyzing the impact on the predictive power of MSWs. We intend to explore more complex and disease-specific arrangements of blood vessels in future work.

4. **Last, while it is stated that this work is on infectious diseases, I would like to see a comment relating this model (or making a suggestion) to heterogeneity in tumors after treatment.**

   This is an important point, as compound diffusion in tumors is thought to contribute to drug resistance and tumor evolution. Based on feedback from the reviewers, we decided to adjust our manuscript to more broadly apply to spatially structured evolving disease populations, including cancer and infectious disease. Connections to cancer were added to the introduction and discussion.

**Reviewer 2**:

1. **In describing the time-variation of MSWs the authors model the variation of drug concentration using a 1-compartment pharmacokinetic model. A description of what parameter values were taken (and why) is missing. Are these based on known values of a particular drug, or typical ranges? A reference here to relevant experimental literature (i.e. with measured $k_{elim}$ and $k_{abs}$) would solidify this result.**

   We thank this reviewer for their consideration of our manuscript. We decided to refocus this work on spatially varying drug concentration. More in-depth analysis of time-varying pharmacokinetics will be explored in a forthcoming paper.

2. **When investigating the spatial dynamics of MSWs the authors derive a steady state solution for the drug diffusion, which they then use to identify different MSW's in space. To obtain this steady state their driving equation (eq. 1) has the property of a constant influx of drug over time. How realistic is this assumption, as opposed to for example a delta spike at t0 or some step function? While the constant flux is clearly necessary to obtain a nonzero steady state, I wonder to what extent this solution is still relevant to the biological problem, considering that in reality to drug would only be introduced for a limited duration. Perhaps if shown that this steady state is reached quickly compared to the duration of the drug injection, there might be a timeframe where it is relevant; but if the quantities of interest are the MSWs, that timeframe should be long enough for selection to act within the different types. Is there some argument for why this constant flux is realistic? Or, is there some way to show (e.g. through numerical solutions out of equilibrium) that the steady state MSW's capture the important behavior?**

   In expanding our work to include stochastic simulations, we implemented a time-varying drug concentration profile using a numerical approximation. We find that, in general, MSWs based on the steady-state drug concentration profile are able to predict certain properties of the evolving population, including spatial heterogeneity and the area occupied by a given genotype. However, the reviewer makes an important point about the relevant timeframe for selection to act on the population. For certain conditions, we find that the spatial structure of MSWs are less predictive because the population distribution has not reached steady-state by the end of the simulation. The particular timeframe necessary for MSWs to drive evolution is ultimately down to properties of the organism and disease state under study. As this work is meant to be a general framework for studying evolution across disease states, including infectious disease and cancer, a disease-specific exploration of the timeframes necessary to make predictions with MSWs is outside the scope of this study, but this comment offers the opportunity for some formal mathematical analysis that could be quite informative – in particular studying the ranges of evolutionary time scales for different organisms/situations. We are eager to continue this study with this new formalism.

3. **Figure 1: In B and C the authors show 3 regimes: "wild type selection", "mutant selection" and "net loss". While the wild type and mutant selection regimes are clearly described as the regime where that type presents the higher growth rate, it is not explained what the "net loss" regime is.**

   The language was updated and clarified and the net loss regime was more clearly defined.

4. **While the authors have done a good job of explaining their own work and results, both in the main text and through clear figures, I found that some background information was lacking, in particular towards readers who are potentially not familiar with all the concepts brought up. I found I needed to read Ref. 7 (Das et al., eLife, 2020) to find definitions of some of the terms used here. While there is of course nothing wrong with referring readers to references for detailed explanations, I believe some minor additions here can greatly improve the readability of the manuscript for readers not already closely familiar with this field.**

   We took care to improve the language and readability of the manuscript. We also added a 'box' to help explain the mutant selection window and fitness seascape terminology.

5. **Line 28: There is no explanation of what is meant by "adjacent genotypes". From ref. 7, I gathered this refers to "genotypes that differ by one mutation". Some definition or explanation here would be appreciated.**

   Indeed, we use 'adjacent' to refer to genotypes that differ by one mutation. We included this definition in our updated manuscript, also now invoking the familiar Hamming distance terminology from information theory.

6. **Line 49: The authors describe pairing each genotype with a random dose-response curve. While this is accompanied by some references, I think the authors could explain a bit more what this conceptually means, i.e. how a resistant strain would enter the tradeoff of taking a lower drug-free growth rate for a higher IC50. Furthermore, the functional form of the dose-response curves is missing, which I strongly feel should be included here (or in the methods section).**

   We added a discussion of tradeoffs and how genotype-specific dose response curves can differ. We also added the functional form of the pharmacodynamic model. We thank the reviewer for bringing this missing information to our attention.

7. **Line 50: "IC50", this technical term could use a definition**

   This term is defined in the updated manuscript.

8. **Line 112: the "1-compartment pharmacokinetic model" could use a reference to point unfamiliar readers toward its derivation.**

   We removed serum pharmacokinetics from the updated analyses.

Reviewer 3:

1. **My primary concern is that the manuscript is simultaneously terse and unfocused, and I am unsure what the central argument or novel insight is.**

   We thank the reviewer for the highly insightful and helpful feedback. We have significantly expanded the analysis included in this work, which we believe strengthens the clarity and impact of the findings. Our central arguments are threefold: 1) we describe how genotype-

specific dose-response curves reveal multiplicitous MSWs in a given system, 2) drug diffusion in tissue reveals the presence of heterogeneous MSWs in space, and 3) the spatial structure of MSWs drives population evolution.

2. **Central terms should be much more carefully defined and should reflect common understanding – for instance, in my experience, 'seascapes' typically refer specifically to time varying fitness landscapes, whereas the manuscript describes temporally varying as well as spatially dependent but stationary conditions. This is not so problematic in and of itself since this is primarily a semantic issue, but it contributes to my sense that the manuscript lacks a clear sense of focus.**

   We tooks strides to improve the clarity of the language in our manuscript. We also included a 'box' that covers fitness seascape and mutant selection window terminology.

3. **While nothing is explicitly wrong, per se, I am left wondering what I am supposed to conclude or learn. As I noted, and as the authors themselves note in the conclusion, many others have demonstrated that space and time are relevant for drug resistance evolution. Therefore, this is not a sufficiently novel point on its own. If instead, for instance, the manuscript focused specifically on temporally varying or spatially varying scenarios alone, and did so in a way that delved into the problem of drug resistance evolution in detail in that specific context, many more interesting and novel insights might be gleaned. Moreover, if the authors presented this in a way that highlighted the generalizability of their results (for instance, by describing what time or length- scales are relevant for drug resistance mutants to emerge with high probability), that would be really great and would certainly be of broad interest.**

   Taking this feedback into account, we removed the serum pharmacokinetics from this manuscript and focused on evolution in spatial gradients. We further explored the generalizability of our results in a sensitivity analysis, where we studied the impact of mutation rate, initial population heterogeneity, and blood vessel geometry on the extent to which MSWs shape evolution.

4. **When it comes to the specifics of the modeling and the assumptions that are made, I see many opportunities for improvement. For instance, dynamics, initial conditions and stochasticity are all important factors in the process of mutation and selection, and yet they are never discussed in detail. Typically, the detailed dynamics of individuals and their positions in the spatial context of a drug gradient will determine the emergence of resistance mutants. Instead, the models discussed are highly simplified and the relevant calculations are limited to ones that are relatively well-known. I am left yearning for more depth in this regard.**

   These are all important considerations. Based on the feedback of the reviewers, we expanded our analyses to include spatial stochastic simulations. These additional experiments allow us to explore more in depth how diffusion shapes mutant selection windows and how spatial mutant selection windows drive population heterogeneity. We explored many of these points raised in this comment in our additional stochastic simulations and analyses.

5. **What happens in more complicated geometries than the ones described? These are almost certainly the relevant scenario in real tissue. What happens when the system is inherently dynamical and spatial, like when there is flow in a blood vessel? This can be highly relevant, as several pathogens are bloodborne rather than tissue resident.**

   While not the focus of our analysis, we performed a simple experiment varying the distance

between blood vessels and found that the predictions made by MSWs are robust to blood vessel geometry. In addition, we focused our analysis on 2-D spatial simulations, which we believe is applicable to both cancer and infectious disease. While certainly relevant, exploring the application of MSWs to bloodborne pathogens is outside the scope of this work.

6. **It is well-appreciated that evolutionary outcomes can be highly contingent on the initial spatial distribution of WT and mutant individuals. How does this play into these types of models? The final and most crucial point is that despite there the presence of highly heterogeneous MSWs in space and time, depending on the relevant length and time scales, this heterogeneity might be 'averaged over' on the relevant length or time scale of growth. Moreover, demographic stochasticity (e.g. genetic drift) might play a much more relevant role than anything related to the deterministic effects of selection. In what parameter regimes should I expect these to be the relevant phenomenology? These are all important considerations for the broader applicability of this type of modeling, and all of these points (especially this last point about the relevant domain of applicability) should be engaged with in earnest in both the analysis and discussion.**

   These are all insightful comments, and we addressed many of these considerations in our sensitivity analysis. We found that the initial distribution of mutants (i.e. initial population heterogeneity) does impact the ability of MSWs to predict final population heterogeneity. Similarly, mutation rate, which drives genetic drift in our model, also affects the extent to which MSWs shape evolution. At moderate mutation rates, selection and mutation work together to drive the population to the genotypes predicted by the MSWs. However, at very high mutation rates, drift dominates over selection and MSWs are no longer predictive. While certainly of interest, we believe a formal investigation of the initial geometry of the population is outside the scope of this work.

7. **The abstract notes that this work highlights the 'utility' of this framework for evolutionary medicine, but I am not sure I see how such an abstract and theoretical model would be utilized in a clinical context. One of the main points of reference in the current manuscript is to Das et al (2018). The Das et al paper primarily discusses the concept of MSWs in a well-mixed setting. Importantly, in Das et al (2018), they explicitly connect this idea to data. What about data in the context of spatially or temporally fluctuating drug concentrations? With the advent of spatially resolved measurements, it is certainly plausible that one might be able to measure some of the relevant spatial gradients that are described. Perhaps the exactly appropriate dataset does not exist yet, but it is sure to come soon. It would be important to address this and to look for relevant data that might constrain the described models or, at the very least, propose what the appropriate experiment might look like given current technologies.**

   We certainly agree that data on compound diffusion in infections and tumors would be crucial for both understanding the generalizability of the MSW model and translating the ideas presented here into the clinic. We also agree that the current clinical utility of the MSW model is limited, and we clarified in the manuscript what we mean by 'utility'. Specifically, we believe that including genotype-specific dose-response curves (which one can measure empirically) and an understanding of spatial drug diffusion can improve the utility of MSWs to predict evolution. In contrast, previous uses of MSWs to predict evolution have focused on the binary resistant-versus-sensitive phenotype model in a well-mixed scenario. Our results demonstrate that MSWs can drive population heterogeneity and predict the relative proportion of different genotypes in the population. In revising the manuscript, we sought to avoid modeling specific disease states and instead maintain a general outlook that may be specifically applied in the future, given the appropriate data. We have expanded the discussion to include these points

and suggest future avenues for experiment and modeling.

8. **Finally, what is the broader relevance? There are certainly many well- established scenarios or conceptual touchstones on which temporal and spatial heterogeneity are a relevant perturbation, but none in particular are highlighted. It would perhaps be most effective to relate these MSW models to other theoretical concepts. One particularly relevant concept is that of fitness valley crossing. It is natural to ask, for instance, what is the simplest scenario in which temporally or spatially heterogeneous drug concentrations will significantly change the probability of fitness valley crossing? And more specifically, by what degree does heterogeneity amplify or restrain fitness valley crossing? This is only one possible example, but it is important that the authors explicitly place this work in a broader context. As it is, it is unclear where this work exactly fits.**

   We thank this reviewer for their insightful suggestions. We have expanded the discussion to relate our work to broader theoretical concepts, including fitness valley crossings and quasispecies theory. We intend to pursue a more formal investigation of the connection between our work and these theoretical concepts in future work.

Our updated manuscript is significantly improved compared to the initial submission, owing largely to the valuable feedback provided by the reviewers. We hope that our resubmission is suitable for publication in *PLoS Computational Biology*, where it will be of interest to a wide range of interdisciplinary scientists.

Sincerely,

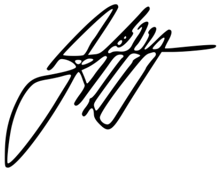

Jacob G. Scott, MD, DPhil (Oxon)
