# Supplementary material for: Diverse mutant selection windows shape spatial heterogeneity in evolving populations
Source: PLoS Comput Biol. 2024 Feb 22;20(2):e1011878. doi: 10.1371/journal.pcbi.1011878 (PMC10914271; doi:10.1371/journal.pcbi.1011878)
Supplement: Attachment — Submitted filename: response.pdf [file pcbi.1011878.s009.pdf]

Taussig Cancer Institute
Cleveland Clinic
Jacob G. Scott, MD, DPhil (Oxon)

Dear Dr. Wodarz and Dr. Komarova,

Thank you for your thoughtful review of our manuscript, "Fitness seascapes promote genetic heterogeneity through spatiotemporally distinct mutant selection windows". In this revision, we have genetically engineered drug-resistant non-small cell lung cancer cells to parameterize our model. We believe that this data increases novelty, validity, and impact of our work.

We respond to each of the reviewers specific concerns below. The reviewer comments are **bolded**. Our responses are directly below each comment.

Reviewer 1:

1. **I remain of the opinion that, in order for the applicability of this work to be demonstrated, the authors should incorporate an example of a particular disease/treatment, or at least provide rigorous connections between their examples (and the parameter values used therein) and particular infection cases encountered in real life.**
   We thank the reviewer for their helpful feedback. We have updated the manuscript to include empirical dose-response curves in a non-small cell lung cancer drug resistance model. We also identified an empirically-derived drug diffusion model in the literature. We used these data to parameterize our agent-based model to demonstrate how mutant selection windows driven by drug diffusion may contribute to drug resistance in cancer. This simulation study provides a connection to a real-world disease using realistic parameters.

Reviewer 2:

1. **Of particular importance is a more direct discussion of the relevant length and timescales on which the discussed phenomenology are relevant. Estimates of length and timescales for the drugs and pathogens/tumors of interest and a comparison to the parameters used in the model are crucial for any downstream applicability and broader interest/relevance.**
   We thank the reviewer for their in-depth comments and insight. With regard to length scale, we have added a discussion of *in vivo* investigation of drug diffusion in the existing literature (lines 167-183). Notably, this analysis suggests that the drug concentration varies considerably across the length scale of of a tumor, thus promoting different mutant selection windows. We go on to investigate this further in our analyses in figures 5 and 6 using empirical cancer growth rate data. These computational experiments involve timescales relevant to cancer therapy. An additional discussion of timescales for the synthetic data is included in lines 132-135.

2. **The authors have made some changes to the manuscript, including adding agent-based simulations. However, these simulations are not described in sufficient detail, nor do they measure the relevant case of de novo evolution of resistance mutants. Rather, they simulate selection on standing variation ("an initial proportion of mutants of 0.1"). This**

latter scenario may have some relevance, but is much less likely, given that resistance is often met with some sort of growth tradeoff, as has been measured, for instance in Pinheiro, et al. Nat Ecol Evol (2021).
We have added additional details for the agent-based simulations in the results and methods sections. We apologize for not adequately explaining the contribution of pre-existing and *de-novo* mutations in our model. We have added additional explanation and analyses, including repeating the experiment in **Fig. 6** with no pre-exhisting heterogeneity (shown in supplemental **Fig. S5**). Our sensitivity analysis also includes the case where there is no pre-existing heterogeneity and all mutations are *de novo*. More detailed information about the HAL simulation software can be found in the original publication (Bravo 2020, PLOS CB). In addition, we believe that fitness costs to resistance, which may be described as a tradeoff, are central to the mutant selection window hypothesis. As we did not sufficiently emphasize this connection in the previous version of the manuscript, we have added additional discussion to Box 1 and the introduction (lines 31-32).

3. **Moreover, the simulations and the subsequent PDE analysis miss key physical processes -- namely that drugs are typically differentially taken up by pathogen vs host cells. Tetracyclines, for instance, are a well-known example of this. When this is the case, the drug concentration field will depend on and evolve with the population of pathogen cells (e.g. the single absorption rate assumption is unlikely to be valid). Therefore drug concentrations typically will not have a steady state concentration profile, and dynamics must be analyzed.**
This is an interesting point – indeed, similar analysis has been explored in cancer in the past (Scott 2016, PLOS CB). This paper is meant to explore a general phenomenon, and therefore any inclusion of specific parameters can only be illustrative. Furthermore, by parameterizing our simulations with an empirically-derived drug diffusion model, we believe that our results are relevant for this system under study. However, we strongly believe that further analysis studying the differential rate of drug uptake and how this may promote diverse MSWs is an interesting and important future direction. We hope that future efforts harness this phenomena to study individual use cases in more detail for scientific questions related to them. We have included comments about differential rates of drug uptake in the discussion.

Reviewer 3:

1. **With regards to the spatial agent-based simulations: if I understand correctly, simulations are initiated with a sparsely populated lattice (line 116-117: "Each simulation was initiated with a random sparsely populated lattice (initial density 0.01 cells per grid position, on average)"), and then allowed to grow to confluency. How realistic is this situation with regards to the systems of interest? For example in cancer, one would expect the the population to already be at "confluency" (i.e. some sort of tissue capacity) when the drug is introduced. In the case of bacteria this may be more realistic, however bacterial cells will generally have some capacity for self-driven motility which likely complicates the picture of selection, as self-assembly and pattern formations can occur (see e.g. 10.1103/PhysRevLett.100.218103 or 10.1073/pnas.1001994107), and cells would be able to move to their ideal position in the gradient. Have you explored how different initial conditions in the population density affect the time evolutions?**
We thank the reviewer for their thorough feedback and kind comments. We agree that careful consideration of initial conditions is important for understanding the validity of our simulations. Based on this feedback, we initialized all simulations as confluent grids, meaning that every grid position is occupied by a cell. Interestingly, initializing the simulations as a circle of radius 10 or a sparse, non-confluent grid did not qualitatively change the results. In

addition, while we did not investigate cell migration, we expect cell migration to increase the relevance of MSWs by promoting admixture and increasing heterogeneity. A full study of cell migration in the context of MSWs is outside the scope of this work, but is an important future direction. We have included some comments on this in the discussion.

2. **130-132: "The difference between the MSW entropy and mean population entropy at $\gamma = 10^{-4}$ is likely due to the fact that the evolving population has not reached steady-state by the end of the simulation, as indicated by the changing cell counts in Fig. 3D." Could this not be tested by running simulations for a longer time, until they reach steady-state? Is there a particular reason simulations were ended at 500h? Given that the MSW prediction for selected genotypes was calculated using a steady state assumption, would it not make sense to compare them with the simulations only when steady state is reached?**
   The initial reasoning behind 500 hours was to maintain a clinically-relevant timescale. By increasing the length of the simulation, one could trivially achieve near-perfect replications of the MSW pattern in the simulated population. However, we increased the length of the simulations in figures 3 and 4 to 1000 hours (roughly 40 days, a timescale relevant to cancer therapy and long-lasting infectious, such a tuberculosis) and found much greater agreement between the MSW and population spatial entropy.

3. **The time dynamics shown in Figure 3D provide great additional insight into the agent-based simulations. It would be interesting to show the MSW population size predictions as additional horizontal lines in the same plots, to see whether the agent based simulations indeed evolve toward the MSW-predicted steady-states.**
   We appreciate the suggestion and have updated figure 3 to include this.

4. **In the section "Sensitivity analysis of mutation rate, initial mutant probability, and blood vessel separation", why introduce "mutation supply = mutation_rate x max population size" as a new parameter, if the max population size is kept fixed? How is this different from studying variation of the mutation rate? It would be clearer to simply use the mutation rate here as parameter of interest.**
   We have updated the sensitivity analysis to include mutation rate rather than mutation supply.

5. **In Figure S1, it would be nice to also see the marginal distribution of the entropy difference for the mutation supply (or mutation rate). I.e. a similar plot as S1C and D, but for the mutation supply.**
   We have updated S1 to include this.

Our updated manuscript is significantly improved compared to the initial submission, owing largely to the valuable feedback provided by the reviewers. We hope that our resubmission is suitable for publication in *PLoS Computational Biology*, where it will be of interest to a wide range of interdisciplinary scientists.

Sincerely,

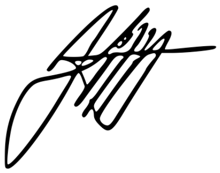

Jacob G. Scott, MD, DPhil (Oxon)
